# Automatic detection and classification of peri-prosthetic femur fracture

**DOI:** 10.1007/s11548-021-02552-5

**Published:** 2022-02-14

**Authors:** Asma Alzaid, Alice Wignall, Sanja Dogramadzi, Hemant Pandit, Sheng Quan Xie

**Affiliations:** 1 School of Electrical and Electronic Engineering, University of Leeds, Leeds, LS2 9JT UK; 2 Collaborates with Institute of Rehabilitation Engineering, Binzhou Medical University, Yantai, China; 3 Trauma and orthopaedics Leeds, Leeds, UK; 4 Department of Automatic Control and Systems Engineering, University of Sheffield, Sheffield, UK; 5 Leeds Teaching Hospitals NHS Trust, Leeds, UK; 6 Leeds Institute of Rheumatic and Musculoskeletal Medicine, Leeds, UK

**Keywords:** Medical imaging, Deep learning, Bone fracture, Surgical planning, Computer aided diagnostics

## Abstract

**Purpose:**

Object classification and localization is a key task of computer-aided diagnosis (CAD) tool. Although there have been numerous generic deep learning (DL) models developed for CAD, there is no work in the literature to evaluate their effectiveness when utilized in diagnosing fractures in proximity of joint implants. In this work, we aim to assess the performance of existing classification systems on binary and multi-class problems (fracture types) using plain radiographs. In addition, we evaluated the performance of object detection systems using the one- and two-stage DL architectures.

**Methods:**

A data set of 1272 X-ray images of Peri-prosthetic Femur Fracture PFF was collected. The fractures were annotated with bounding boxes and classified according to the Vancouver Classification System (type A, B, C) by two clinical specialists. Four classification models such as Densenet161, Resnet50, Inception, VGG and two object detection models such as Faster RCNN and RetinaNet were evaluated, and their performance compared. Six confusion matrix-based measures were reported to evaluate fracture classification. For localization of the fracture, Average Precision and localization accuracy were reported.

**Results:**

The Resnet50 showed the best performance with $95\%$ accuracy and $94\%$ F1-score in the binary classification: fracture/normal. In addition, the Resnet50 showed $90\%$ accuracy in multi-classification (normal, Vancouver type A, B and C).

**Conclusions:**

A large data set of PFF images and the annotations of fracture features by two independent assessments were created to implement a DL-based approach for detecting, classifying and localizing PFFs. It was shown that this approach could be a promising diagnostic tool of fractures in proximity of joint implants.

## Introduction

**Fig. 1:**
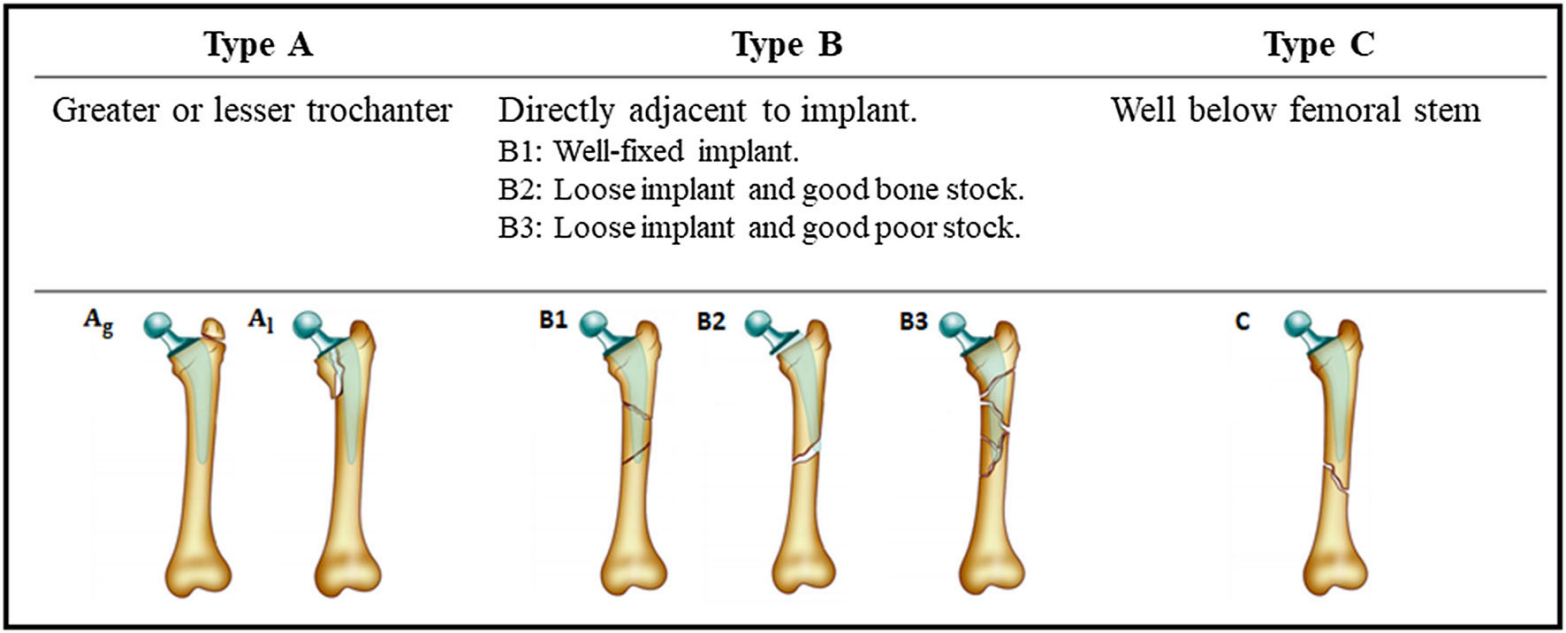
The classification of PFFs according to VCS [30]

In 1991 it was suggested that total hip replacement (THR) may be the operation of the century that can provide excellent pain relief and an improved quality of life for patients with severe arthritis [18]. With a growing elderly population, the rates of THRs is increasing (approximately 90,000 procedures per year in the UK) [36] accompanied by an unavoidable rise in associated post-operative complications such as Peri-Prosthetic Femur Fractures (PFFs) that occur in 3.5% of patients who undergo THR [1]. Following a primary THR, PFF accounts for 10.5% of revision hip arthroplasties [36] and it is predicted that 4.6% of THR patients can be affected by PFF [1]. PFFs are usually caused by low energy falls in elderly patients, but can also be due to implant loosening, osteolysis or stress from an adjacent implant. The assessment and management of PFFs relies on a clinical assessment of the patient, prior operation notes on the joint implant and surgical approach taken, and the fracture image to assess the fracture characteristics and the implant for loosening and osteolysis [27]. The management of PFF varies from non-operative treatment to open reduction and internal fixation (ORIF) to revision of the prosthesis [19]. The Vancouver Classification System (VCS) is commonly used to characterise these fractures and guide the subsequent surgical management (see Fig. 1). VCS considers three main fracture features: fracture location, implant loosening and bone quality [4].

Optimal management of PFF patients is guided predominately by the associated radiographic appearance, however it has been found that $90\%$ of PFF radiology reports do not include all relevant radiographic features. This may lead to a delay in diagnosis and incorrect treatment strategy and, ultimately, delayed surgery [24].

Current efforts in computer aided fracture diagnosis focus on the detection of fracture only. However, the reported work exclude the fracture cases with a prosthesis when designing automatic fracture detection systems [34]. One of the essential tasks that computer-aided diagnosis (CAD) for fracture needs to address is identifying the type of the fracture. A few existing fracture diagnosis techniques are focused on specific regions of the bone, for example, proximal femur [14]. In case of PFFs, the location of fracture varies significantly and can be in different femur regions.This significantly increases the variation between images and complexity of the detection problem.

**Fig. 2:**
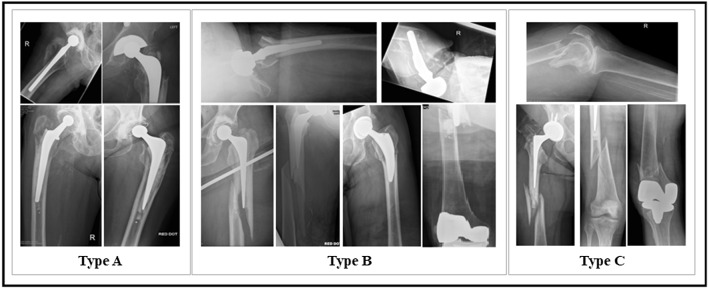
Illustration of the quality of X-ray images, fracture line appearance and the high variability of PFFs in X-ray images; image view, implant type and captured bone part

The detection, localization and classification of the fractures from X-ray images can face one or more of the following issues (refer to Fig. 2): (1) poor quality of X-ray images due to noise and low contrast. (2) fracture lines often hard to identify. (3) significant variations in fracture location, fracture pattern, image views and, specifically for PFFs, additional variations related to implant type and capturing locations.

For this work, we annotated a large dataset of PFF images with bounding boxes and fracture classes. In addition, we evaluated different deep learning approaches to identify, classify and localize PFFs from X-ray images using the VCS to assist orthopaedic surgeons in fracture management that can ultimately enhance patient outcomes.

The rest of the paper is organized as follows: the second section summarises the related work of fracture diagnosis. The methodology is presented in the third section. Followed by the experiments and result discussions. Finally, the last section provides conclusion and future work.

## Related work

The huge development of machine learning techniques made a major impact on improving the detection and diagnosis of different diseases such as Lung nodule detection in the chest [6, 10], mass detection [38] and mass classification into benign or malignant [9]. A collection of research and methods on CAD in medical images can be found in [8, 22]. Compared to these developments, techniques for automatic diagnosing bone fractures are scarce [15].

The existing methods for automatic image analysis of bone fractures are based either on hand-crafted features or learning relevant image features.

### Feature-based method

The early work on fracture detection and classification focused on a typical machine learning framework that generally consisted of pre-processing, feature extraction and classification steps. For the pre-processing step, many low level pixel-processing methods such as noise reduction and segmentation were used to obtain the region of interest (ROI). Using ROI, various features can be extracted for classification of bone fractures. The feature types can be texture analysis [5, 11, 12], combination of texture and shape features [35] or digital geometry of the extracted fracture points [3]. For the classification step, the fusion of multiple classifications resulted in improved fracture prediction [23, 35] when compared to using a single classification approach [5] .

The hand-crafted feature-based approaches require a prior knowledge of the specific feature to be extracted which affects their generalization ability. In addition, most of these methods rely on a prior segmentation of the bone, the process that typically lacks accuracy in extracting bone contours. Modeling and representing a bone fracture is complex due to a large number of parameters involved but it could be learned from a large set of relevant image data.

### Deep learning-based method

The recent developments of deep learning techniques have overcome some limitations of traditional feature-based approaches. Convolutional neural networks (CNN) have demonstrated the ability to detect fractures by performing the binary classification task (fracture or normal) in different anatomical regions, such as hip [7], pelvis [37], wrist [21], spine [29] and ankle [17]. Imagenet [16, 26, 37], or a similar dataset (bone x-ray images) [7, 21] can be used to pre-train a network in order to improve accuracy of classification. Moreover, it is illustrated in several studies that cropping the ROI and feeding it to the network increases the classification accuracy [14, 37]. Combining hospital process variables such as hospital department, scanner model, patient demographic information (age, gender, body mass etc.) can further improve fracture prediction outcome when compared to using just X-ray images of the fracture [2].

All the above studies focus on a specific part of the fractured bone, e.g. proximal femur [14], and do not consider more diagnostically complex fractures close to joint implants, see Fig. 2. There is a wide range of fracture types with different visual patterns at different anatomic locations. Additionally, there is variability in the X-ray images in terms of capturing different parts of the bone for the same fracture type. In contrast to hip or other aforementioned fractures, which are located at a specific position, for example the femoral neck, PFFs can be located anywhere on the femur, around or below the implant. This increases the complexity of image pattern analysis and makes the extraction of a ROI based only on the bone anatomy more difficult.

Therefore, we considered in depth evaluation of a deep learning-based approach to tackle diagnosis of PFFs as both a detection of the presence of the fracture (binary classification ’fracture, normal’) and a classification of the fracture according to the VCS.

**Fig. 3:**
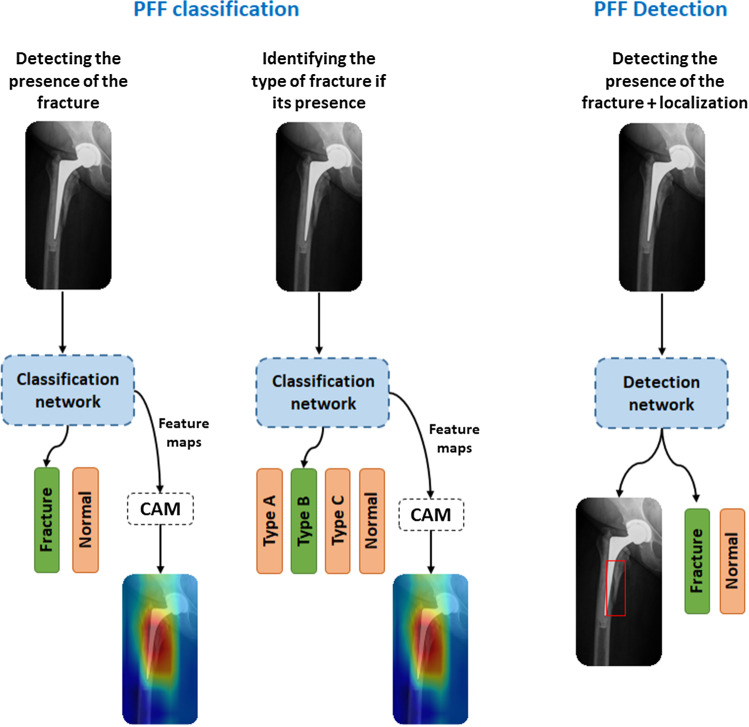
PFF classification approach: the examined classification network are (ResNet, DenseNet, VGG and Inception). The object detection network: FasterRCNN and RetinaNet

## Method

We developed a CAD tool based on CNN and systematically explored different model architectures. In this direction, two approaches were examined: PFF classification (‘PFF classification’ Section) and PFF detection, which combines both the classification and localization (‘PFF detection approach’ Section). Figure 3 presents a general overview of these approaches.

### PFF classification

Given a set of X-ray images $I \in R^{H\times W}$, our goal was to train a classification model $\textit{f}(\cdot )$ in order to specify a class label $y \in C$ for each image ($I_i$). Two sets of class labels were considered - $C \subset {\{fracture, normal\}}$ for detecting the presence of a fracture and $C \subset {\{Type A, Type B, Type C, normal\}}$ for categorization of the fracture. The classification model can be defined as:

1 $$\begin{aligned} y = f(I;w_f) \end{aligned}$$

Where *I* is the X-ray image and $w_f$ is the model parameters. The function *f* is approximated using a CNN optimized to minimize the cross-entropy loss function:

2 $$\begin{aligned} \ell _{class}= -\sum _{j \in C}y_{j,c}log(y_{j,c}). \end{aligned}$$

#### Visualization of PFFs

To visualize the fracture region, we used the Class Activation Map (CAM) [39] method, which generates a weighted activation map for each image. This identifies a region that a classification model is focusing on. The CAM method depends mainly on the global average pooling layers which are added after the last convolutional layer of the network to create the spatial average of the feature map of each image unit. Given an image, let $f_k(x,y)$ denote the activation of unit *k* in the last convolutional layer at a spatial location (*x*, *y*). Then, the result of average pooling for unit *k* is $\sum _{x,y}f_k(x,y)$ and the class activation map for class *c* for a spatial element is defined as:

3 $$\begin{aligned} M_c(x,y) = \sum _{k}w_{k}^{c}f_{k}(x,y) \end{aligned}$$

Thus, the class score $S_c= \sum _{x,y}M_c(x,y)$. The $M_c(x,y)$ shows the importance of the activation at (*x*, *y*) resulting in the image classification to a class *c*.

To highlight salient features in the X-ray image that discriminate abnormality, the CAM is up-scaled to the image dimension and overlays the image.

### PFF detection approach

In the PFF detection approach, we attempted to classify and localize the PFFs using image labels and vertices of a fracture bounding box in a fully supervised fashion. The following sections describe two object detection models: Faster RCNN [28] and RetinaNet [20].

#### Faster RCNN

Faster R-CNN is a two-stage object detection model: Region Proposal Network (RPN) and Fast R-CNN. Both stages share the same backbone network, which outputs the feature map of the input X-ray image.

*RPN* is a fully convolutional network responsible for generating region proposals with various scales and aspect ratios which are used by Fast R-CNN for fracture detection. The RPN applies the concept of attention to tell the (Fast R-CNN) where to look. First, a sliding window with a size $n\times n$ is passed through the feature maps to generate *K* anchors with a different size and aspect ratio for each location. For each pixel, the network checks whether these *K* anchors contain an object (fracture) or not. Therefore, for each anchor, a feature vector is extracted and fed to two fully connected layers. The first one is a binary classifier that computes the objective score, i.e if the area includes an object (fracture) or not. The second one returns the bounding box as region proposals.

*Fast R-CNN* The feature maps from the backbone network and the resulted region proposals are fed to the ROI pooling layer. The ROI pooling layer splits each region proposal into grid cells and applies a max pooling operator to each cell to return a single value. The output feature vector is defined by all values from all these cells. The feature vector is then passed to the fully connected layer which is divided into two sub-networks: the softmax layer that predicts class scores and the regression layer that predicts the bounding box coordinates.

#### RetinaNet

RetinaNet is a one stage object detection model, which consists of three sub-networks: a backbone network, a Feature Pyramid Network (FPN), and Fully Convolutional Networks(FCNs).

*Backbone network* computes a feature map of the input X-ray image.

*FPN* is used to construct a rich multi-scale feature pyramid from a single scale input image. The structure of the pyramid consists of two pathways: bottom-up and top-down. The first pathway computes a feature hierarchy by using the feature activation output of each residual block. The high level feature maps are considered in the top-down pathway by up-sampling spatially coarser feature maps from the higher pyramid levels.

*FCNs* This sub-network includes two FCNs. The first FCN performs the classification task (fracture/ no fracture), while the second one performs the bounding box regression (localization of the fracture).

RetinaNet uses a focal loss function to resolve the class imbalance problem between the background and foreground in the detection scenario. Thus, the standard cross entropy loss has been modified to the following:

4 $$\begin{aligned} FL(p_t) = -\alpha _{t} (1 - p_{t})^{\gamma } log(p_t). \end{aligned}$$

where $$ p_t = {\left\{ \begin{array}{ll} p &{} \text {if y=1}\\ 1-p &{} \text {if otherwise} \end{array}\right. }$$

$\gamma$ is a tuning focusing parameter $(\gamma \ge 0)$

## Experiments

### Dataset collection and preparation

The experiment was approved by the Healthcare and Medicine Research Ethics Committee at the University of Leeds (MREC 19-005). The dataset of PFF images was collected at multiple trauma centers in the United Kingdom. Overall, 607 anonymised patient data were collected with a total of 2544 X-ray images. To establish a ground truth classification and detection for the images, two clinical experts participated in image annotations and provided class labels and fracture bounding boxes. 59% of the images were annotated by both experts and the rest with a single annotation.

For each patient, we collected either a lateral or an anterior-posterior (AP) image or both. The images included either a partial region of the femur, the full femur or the pelvis with both femurs. The last type of image was split into two, containing one femur each. The images were of various scales, orientations and implant types. Images for each patient included an X-ray after THR surgery (representing the normal cases) and an X-ray containing the fracture. The images were annotated by class labels (Type A, Type B, Type C and normal). The fracture images were further annotated by a bounding box around the fracture, i.e the coordinates of the minimum and maximum corners of the rectangle. For annotations we used Microsoft Visual Object Tagging Tool (VOTT).

*PFF classification* For the classification task, both binary classification (fracture vs normal) and multi-classification (Type A, Type B, Type C and normal) were considered. For binary classification, 1272 images with a fracture and 1272 images without a fracture (normal) were used. For the multi-classification task, the dataset consisted of: 375 normal, 88 Type A, 375 Type B and 378 Type C images. The number of images of Type B was very high (63% of the fracture images) when compared to the other types (A and C). Therefore, we randomly excluded 431 images from Type B. For both tasks, the dataset was divided into two parts: training and validation, with the ratio $75\%:25\%$, respectively.

**PFF detection** In this experiment, we focused on detecting the fracture region and considered two classes: fracture and background (normal). The same dataset of fracture images in the binary classification experiment was split into the training and validation sets.

### Model architectures and implementation details

All the models were trained on a Windows machine equipped with 8 GB RAM, Intel(R) Core(TM) CPU @ 3.00 GHz and GeForce RTX 2080 graphics card.

*PFF classification* For classification tasks, we compared different network architectures (ResNet50 [11], VGG [32] , DenseNet161 [13], Inception [33]) that were pre-trained on ImageNet. Each network was trained on X-ray images down-sampled from the original size to 224 $\times$ 224 px, except Inception model which accepts 299 $\times$ 299 px. The classes included ‘normal’ and the categories of VCS. Data augmentation techniques such as flipping, rotation and scaling were used. The CAM is used on top of each model to visualize the fracture region.

For optimization, we used Stochastic Gradient Descent (SGD). All the models were trained until convergence (100 epoch). The batch size was 8, momentum 0.9 and learning rate was set to 1 $\times 10^{-2}$.

*PFF detection* Both models were trained and validated using different image resolutions. For the backbone network, ResNet50 was used in both object detection models and the optimization was performed using SGD. All the models were trained until convergence (100 epoch). The batch size was 2, momentum 0.9. We used the default anchor configuration and non-maximum suppression with IoU 0.7. The learning rate was set to 1 $\times 10^{-2}$ on Faster R-CNN and 5 $\times 10^{-2}$ on RetinaNet.

### Evaluation settings

To evaluate the classification results, we used the standard metrics derived from Confusion Metrics: accuracy, precision, recall (sensitivity), specificity, F1 score and AUC-ROC curve. The classification accuracy determines the percentage of the correct estimated class (fracture/ no fracture) in respect to the ground truth. The precision measures the proportion of predicted fracture images that were actually correct. The recall measures the proportion of actual fracture images that were identified correctly. Specificity measures the proportion of predicted normal images that were actually correct. The ROC curve is a probability curve that plots the true positive rate against false positive rate at various threshold values and the AUC is used to measure the ability of the classifier to differentiate between the classes.

In addition, for a qualitative analysis of clinical applicability of the classification model, we visualized the part of the X-ray image that contributes more to the prediction as explained in Visualization of PFFs section. For the object detection task, we measured the localization accuracy which considered the tested image as correct if both predicted classes and the bounding box were correct. The correct bounding box was defined using the Intersection Over Union (IOU) measure which computes the overlap area between the ground truth box and the predicted box over the area of union of them. The predicted bounding box was considered as correct when IOU $\ge 0.5$. In addition, we reported the precision, recall and Average Precision (AP).

**Fig. 4:**
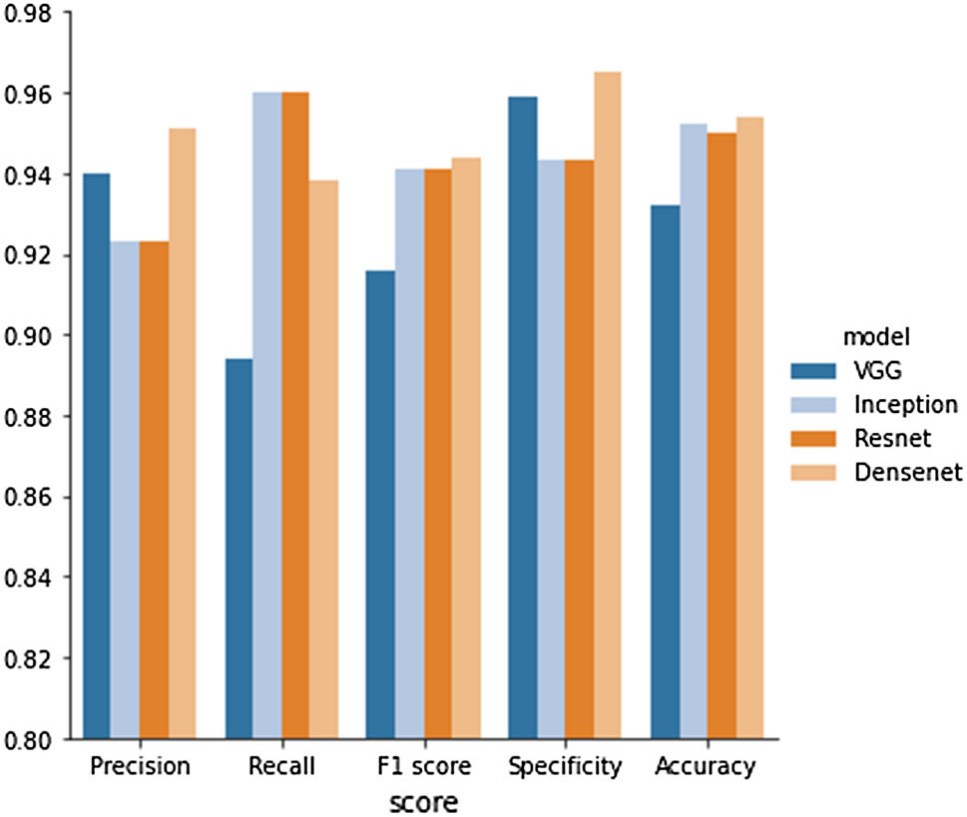
Comparison of the performance of Fracture/ no fracture classification

**Fig. 5:**
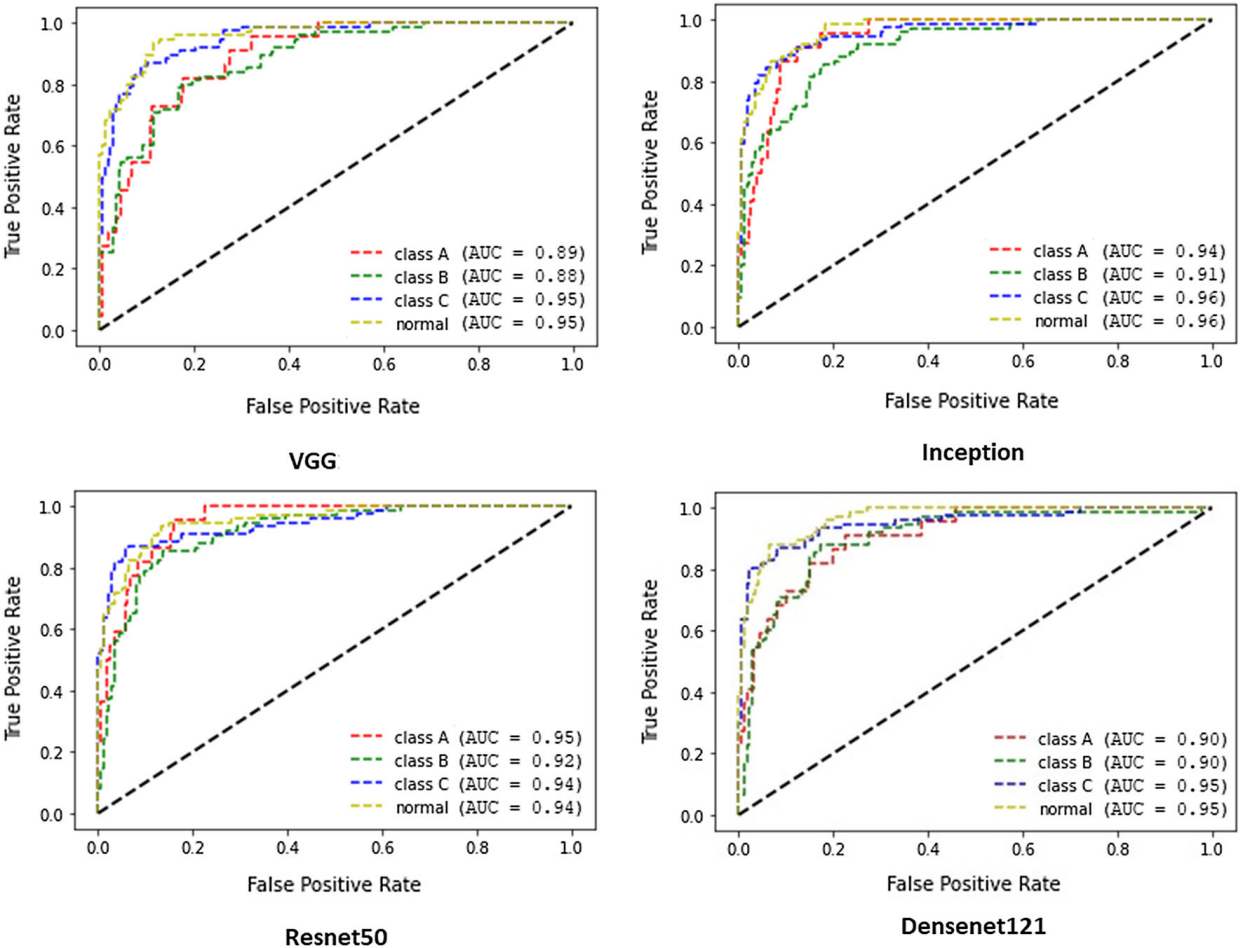
ROC curves for Fracture Types A,B and C and Normal class for each classification model

**Table 1:** Precision, recall, F1-score and accuracy of PFFs classification. The highest metric average values across the four models are highlighted in bold for each metric

	VGG					Inception					Resnet50					Densenet				
	A	B	C	Normal	Avg.	A	B	C	Normal	Avg.	A	B	C	Normal	Avg.	A	B	C	Normal	Avg.
*Full image*																				
Precision	0.50	0.71	0.83	0.80	0.71	0.50	0.77	0.83	0.85	0.73	0.71	0.76	0.86	0.83	**0.79**	0.63	0.78	0.81	0.84	0.76
Recall	0.32	0.71	0.84	0.88	0.69	0.59	0.65	0.87	0.88	**0.75**	0.45	0.83	0.86	0.84	0.74	0.55	0.71	0.87	0.88	**0.75**
F1 Score	0.39	0.71	0.84	0.84	0.69	0.54	0.71	0.85	0.86	0.74	0.86	0.79	0.86	0.83	**0.83**	0.59	0.74	0.84	0.93	0.77
Specificity	0.97	0.87	0.92	0.91	0.92	0.94	0.91	0.92	0.93	**0.93**	0.98	0.89	0.94	0.93	**0.93**	0.97	0.91	0.91	0.91	0.92
Accuracy	0.91	0.82	0.90	0.90	0.88	0.91	0.84	0.90	0.92	0.89	0.94	0.87	0.91	0.90	**0.90**	0.93	0.85	0.90	0.91	**0.90**
*Manual ROI*																				
Precision	0.62	0.67	0.79	0.83	0.73	0.53	0.73	0.85	0.83	0.73	0.67	0.70	0.88	0.82	**0.77**	0.57	0.72	0.90	0.86	0.76
Recall	0.36	0.75	0.81	0.83	0.69	0.41	0.77	0.86	0.83	0.72	0.45	0.83	0.83	0.80	**0.73**	0.36	0.83	0.83	0.89	**0.73**
F1 score	0.46	0.71	0.80	0.83	0.70	0.46	0.75	0.85	0.83	0.72	0.54	0.76	0.85	0.81	**0.74**	0.44	0.77	0.86	0.88	**0.74**
Specificity	0.98	0.84	0.91	0.93	0.91	0.96	0.88	0.93	0.93	0.92	0.98	0.85	0.95	0.93	**0.93**	0.97	0.86	0.96	0.94	**0.93**
Accuracy	0.92	0.82	0.88	0.90	0.88	0.92	0.85	0.91	0.90	0.89	0.93	0.84	0.91	0.89	0.89	0.92	0.85	0.92	0.92	**0.90**

## Results

### PFF classification

Two classification experiments of PFFs were evaluated—binary classification to distinguish between fracture and no fracture X-ray images and classification according to VCS.

In the binary classification task, we evaluated different network architectures (Inception, VGG, ResNet50 and DenseNet161). Figure 4 presents the accuracy, precision, sensitivity (recall), specificity and F1 score for each model of binary classification. The Densenet161, Resnet50 and Inception models provided similar accuracy—around $95\%$. Both Resnet50 and Inception models detected 96% of fracture images correctly. As no current automated methods for PFF detection has been found in the literature, this result could be considered the state of the art. Compared to Miao et al. [25], method for detecting fractures in femur with no presence of implants, our results outperform their stated accuracy of fracture detection (91%).

In multi-classification task, after excluding uncertainty labels, the dataset consists of 1216 studies. The dataset presents class imbalances ($7\%$ TypeA, $31\%$ TypeB, $31\%$ TypeC, $31\%$ normal), therefore, AUC-ROC curve as well as standard metrics derived from confusion metrics were reported (see Fig. 5 and Table 1). We analysed the performance of the aforementioned models in each class. It is clear from Table 1 that the performance decreased when the task became more difficult (multi-classification), especially when we considered the recall score. The performance of correctly classified fracture types was reduced by $10\%$.

To evaluate the effect of cropping the ROI (femur) on the performance of the classification, we considered two approaches: (1) using the full image as input. (2) using the cropped ROI (femur region) as input. The existing fracture diagnosis approaches have achieved better performance when a cropped ROI was utilized. For instance, [14] applied an ROI cropping method to localize the proximal femur region in a pre-processing step of fracture classification. This allows the variety between the images to be reduced and the model to learn the shape of the proximal femur. However, in PFFs the fracture can be located at different regions of the femur. In addition, the analysed X-ray images contained different regions of the femur which further increased the image variation. Therefore, the classification of PFFs using a femur region as a ROI had a similar accuracy as when the full image was used as shown in Table 1.

Regarding the average AUC for the classification of the fracture, Resnet50 and Inception performed best (See Fig. 5). Broken down for individual results, the most precise detection of Type A and Type B fractures was accomplished by Resnet50 (0.95). For detection of TypeC fracture, Inception net was the best model.

Overall, Resnet50 provided the best performance of the PFF classification types with an average accuracy of $90\%$. On the other hand, when focusing on Recall metric to measure the performance of the correctly classified fracture cases, Resnet50 was able to classify $45\%$ of Type A, $83\%$ of Type B and $86\%$ of Type C images. The low performance in classification of type A could be related to the smaller number of this type of images. In addition, distinguishing between Type B and C resulted in a slightly lower performance, $10\%$ of Type C fractures were classified as Type B which is not surprising considering that these two types look similar in some cases.

The majority of the previous CAD systems for fracture analysis focuses on abnormality detection. Few works have been introduced to classify the fracture types such as [14]. Their work focuses on a specific region of femur and requires cropping of the femur proximal area before analysis. Our method did not require this stage and achieved similar performance accuracy.

When using CAD tools, it is important to visualize the region of interest in order to support the decision making process. In addition, it is important to base the evaluation on a correct analysis of fracture features. Therefore, we used CAM method to highlight the region that the model focuses on to predict a class type. Figure 8b presents some results of classification of PFF images using Resnet50 model.

### PFF detection

**Fig. 6:**
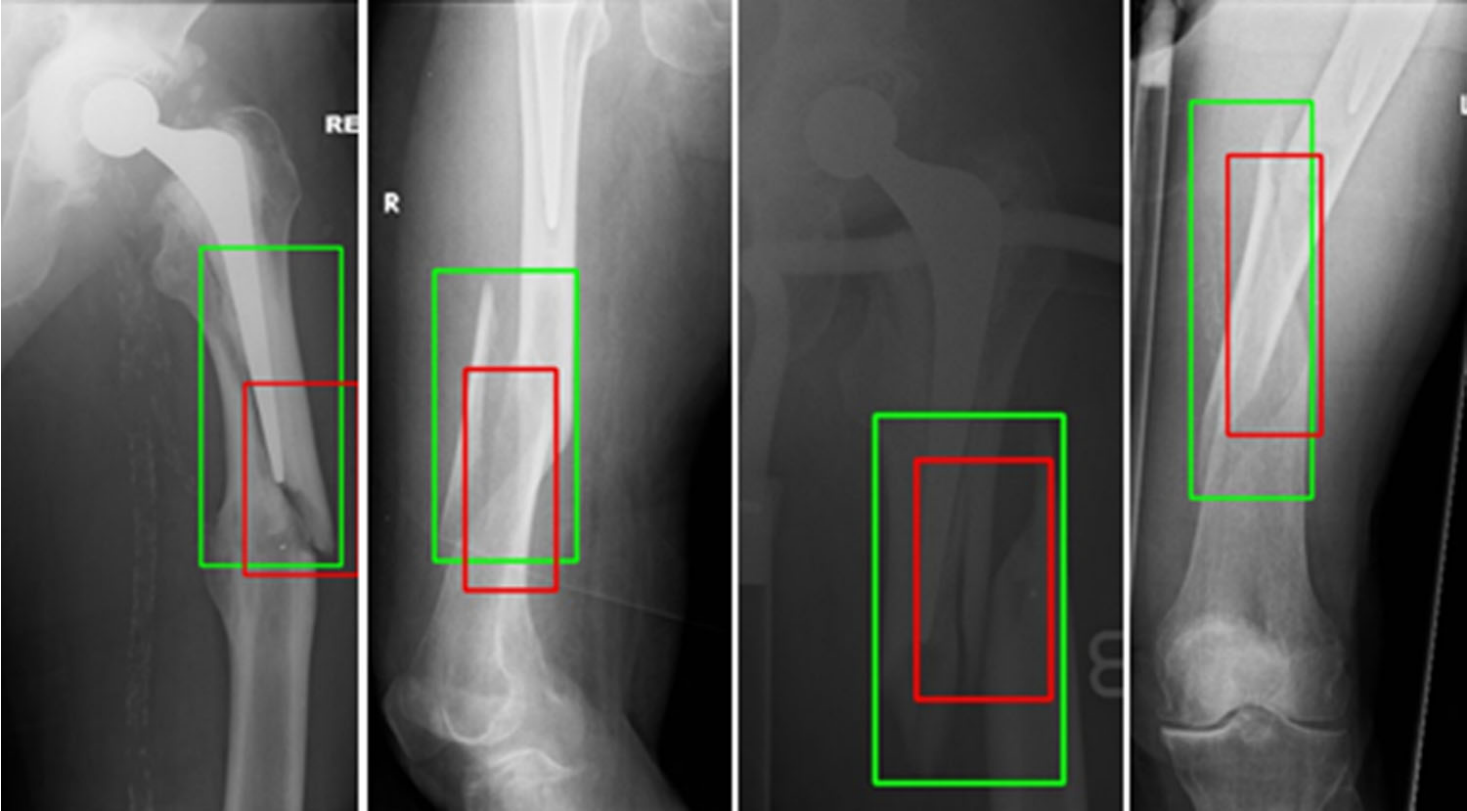
CAM-based fracture localization. (green box is the ground truth and red is the CAM result)

The CAM method provides only an approximate localization of a fracture because it tends to concentrate on the most discriminate region of the fracture. Weakly supervised object detection approach, such as the CAM based method we used, utilized image level labels only to classify and localize fractures in the images. The fully supervised object detection approach used both image labels and fracture region annotations in the training phase. Therefore, the performance gap between the two approaches is still large [31] (Fig. 6).

**Fig. 7:**
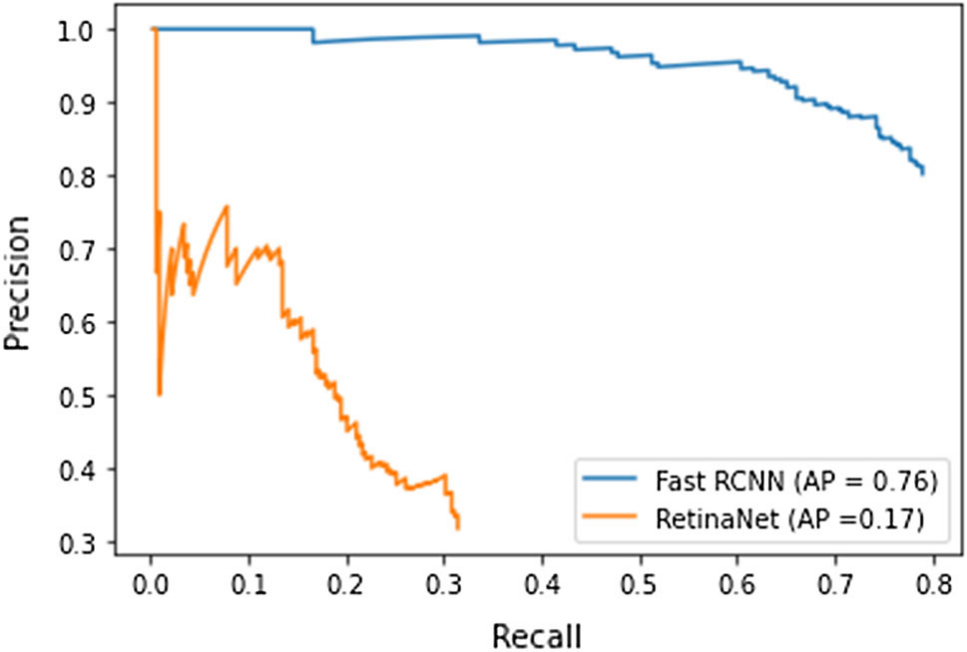
Precision-Recall curve for Faster RCNN and RetinaNet

The two state-of-the-art object detection models that we evaluated are: Faster R-CNN and RetinaNet. Table 2 presents the precision, Recall an accuracy obtained by the two detection models (Faster RCNN and RetinaNet). As can be seen, Faster RCNN provides the best performance. The recall results, in Table 2, showed that both model were able to detect majority of ground truth images. The precision results showed that 80% of these detections were correct using Faster RCNN, while 31% only using RetinaNet. The localization accuracy of the Faster R-CNN was $78\%$. It reached AP value of 76 in contrast to RetinaNet which provide very low AP (see Fig. 7).

**Fig. 8:**
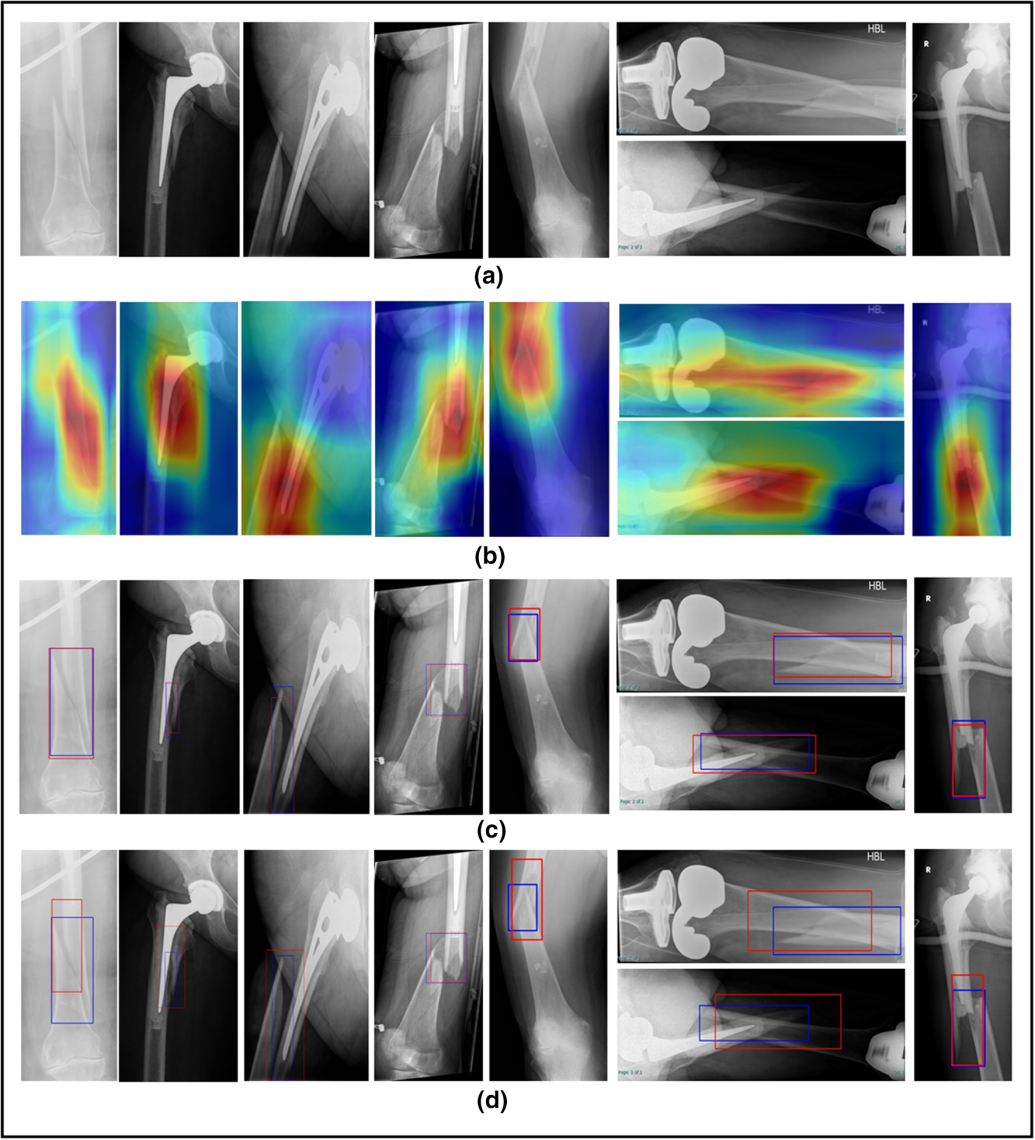
(**a**) the original x-ray images. (**b**) Resnet50 classification results with the CAM. The heat map color range from blue (minimum) to red (maximum). (**c**) fracture bounding box results of Faster RCNN (**d**) fracture bounding box results of RetinaNet (blue is the ground truth and red is the predicted box)

Figure 8c and d shows some examples of the predicted fracture location using Faster R-CNN and RetinaNet.

The localization of PFF fractures in X-ray images can be difficult to narrow to the boundary box so the box may include multiple anatomical regions. This increases the ambiguity of the bounding box. However, the Faster R-CNN provides promising results for PFF localization (Fig. 8).

## Conclusion

There are increasing cases of PFFs in the elderly population, associated with the increase in rates of THR. An accurate clinical diagnosis for this type of fracture is essential for taking a correct treatment approach and, subsequently, for the overall clinical patient outcome. Unlike existing techniques developed for fracture detection, this work concentrates on a framework for automated diagnostics of fractures in the proximity of joint implants (hip). Our in depth evaluation of different methods demonstrated that Resnet50 is able to detect PFFs with an accuracy of $95\%$, and classify fracture type with an accuracy of $90\%$ . CAM method provided an approximate visualization of the fracture region. However, Faster RCNN predicted a narrower bounding box of the fracture region with a localization accuracy of $78\%$.

**Table 2:** Precision, recall, and accuracy of PFFs detection (classification and localization)

	Faster RCNN	RetinaNet
Precision	80	31
Recall	98	97
Accuracy	78	31

The future work will consider more complex approaches to improve the accuracy of the classification of the fracture types, by incorporating additional information based on the expert surgeon’s diagnostic patterns such as identifying regions and features they pay particular attention to. Defining attention maps as ROI will enhance the features from fracture related regions while preserving the global feature from the X-ray image. It is hoped that this methodology will help the clinicians and thereby patients in improving the diagnosis of PFF, thereby reducing variation in the existing practice.
